# Polyphenol Rich *Forsythia suspensa* Extract Alleviates DSS-Induced Ulcerative Colitis in Mice through the Nrf2-NLRP3 Pathway

**DOI:** 10.3390/antiox11030475

**Published:** 2022-02-28

**Authors:** Limin Chao, Jin Lin, Jing Zhou, Hongliang Du, Xiaoli Chen, Mengjie Liu, Qian Qu, Weijie Lv, Shining Guo

**Affiliations:** 1 College of Veterinary Medicine, South China Agricultural University, Guangzhou 510642, China; chaolimin@stu.scau.edu.cn (L.C.); linjin@stu.scau.edu.cn (J.L.); zhouj2020@stu.scau.edu.cn (J.Z.); 18819268540@stu.scau.edu.cn (H.D.); xiaolichen@stu.scau.edu.cn (X.C.); mengjieliu@stu.scau.edu.cn (M.L.); qianqu@stu.scau.edu.cn (Q.Q.); 2 Guangdong Technology Research Center for Traditional Chinese Veterinary Medicine and Nature Medicine, Guangzhou 510642, China

**Keywords:** *Forsythia suspensa*, ulcerative colitis, antioxidant, pyroptosis, metabolomics

## Abstract

This study systematically evaluated the effect of *Forsythia suspensa* extract on dextran sodium sulfate (DSS)-induced ulcerative colitis (UC) and determined its mechanism of action. The results showed that *Forsythia suspensa* extract significantly inhibited DSS-induced UC in mice. In vivo mechanistic studies revealed that *Forsythia suspensa* extract relieved the symptoms of colitis by enhancing antioxidant activity and inhibiting pyroptosis. Further in vitro experiments on the mechanism of *Forsythia suspensa* showed that it reduced the level of reactive oxygen species (ROS) in J774A.1 cells. We found that *Forsythia suspensa* extract enhanced cellular antioxidation activity and inhibited pyroptosis. After silencing NLRP3, it was found to play an important role in pyroptosis. In addition, after Nrf2 was silenced, the inhibitory effect of *Forsythia suspensa* extract on cell pyroptosis was eliminated, indicating an interaction between Nrf2 and NLRP3. Metabonomics revealed that *Forsythia suspensa* extract significantly improved metabolic function in colitis mice by reversing the abnormal changes in the levels of 9 metabolites. The main metabolic pathways involved were glutathione metabolism, aminoacyl-tRNA biosynthesis and linoleic acid metabolism. In conclusion, we found that *Forsythia suspensa* extract significantly alleviated DSS-induced UC injury through the Nrf2-NLRP3 pathway and relieved metabolic dysfunction.

## 1. Introduction

Ulcerative colitis (UC) is a common nonspecific inflammatory disease characterized by chronic inflammation and ulcers in the lining of the rectum and colon. The clinical manifestations include pain, weight loss, bloody stools and intestinal mucosal ulcers [1,2]. The development of UC involves many factors, such as microenvironmental factors, genetic factors, an abnormal immune response, and destruction of the colonic barrier [3,4]. Pyroptosis is a form of programmed cell death. Excessive release of IL-1β during pyroptosis can lead to severe acute and chronic inflammatory diseases [5]. Many studies have found that inhibiting pyroptosis signalling can alleviate experimental colitis. Cholecalciferol cholesterol emulsion alleviates the damage caused by experimental colitis by inhibiting pyroptosis [6]. Knockdown of Gasdermin E (GSDME) can attenuate ATP-induced pyroptosis in mouse macrophages [7].

*Forsythia suspensa (Thunb.) Vahl* is a well-known traditional medicine in Asian countries such as China, Japan and Korea [8]. The fruit of *Forsythia suspensa*, which is collected in August, is called Qingqiao, and the mature fruit of *Forsythia suspensa*, which is collected in October, is called Laoqiao. Recent studies have found that *Forsythia suspensa* has a variety of anti-inflammatory, antioxidant, antibacterial and anti-allergic effects. *Forsythia suspensa* extract was observed to alleviate colon tissue damage in mice with UC [9]. *Forsythia suspensa* extract also exerts a protective effect against the oxidative stress induced by diquat [10]. Previous studies have found that the anti-inflammatory and antioxidant effects of *Forsythia suspensa* extract are related [11,12,13]. Polyphenol compounds exert important biological activities [14]. Recent studies have found that polyphenol compounds have a certain effect on inflammation and oxidative stress [15,16,17].

Currently available drug treatments can only increase the duration of the clinical remission period and slow down the destructive and progressive disease process [18]. Although it has been confirmed that *Forsythia suspensa* extract alleviates damage in mice with colitis, the mechanism of action remains to be determined [9]. Therefore, this study aimed to explore the mechanism by which *Forsythia suspensa* extract ameliorates dextran sodium sulfate (DSS)-induced UC in mice and to investigate whether the mechanism is related to antioxidation and pyroptosis. To further elucidate on how *Forsythia suspensa* extract affects the colon, we analyzed the changes in the metabolic characteristics of colitis mice after *Forsythia suspensa* extract treatment and identified biomarkers and potential therapeutic targets through metabolic pathway analysis.

## 2. Materials and Methods

### 2.1. Drugs

*Forsythia suspensa* (Qingqiao) in this study was collected in Henan Province, China, in August (Anguo Yuzheng Chinese Medicine Pieces Co., Ltd., Hebei Province, China). *Forsythia suspensa* powder was produced with a Chinese medicine pulverizer and filtered through a 60-mesh sieve. The ultrasonic extraction conditions were as follows: temperature 53 °C, ethanol concentration 62%, time 0.72 h, material-to-liquid ratio 38 mL/g. The supernatant was collected and concentrated by evaporation under reduced pressure. The *Forsythia suspensa* extract was stored in a freezer at −20 °C.

### 2.2. Experimental Animals

Fifty 7-week-old male C57BL/6J mice were purchased from Beijing Vital River Laboratory Animal Technology Co., Ltd. (Beijing, China). Under the guidelines of the Laboratory Animal Center of South China Agricultural University, the animals were raised in specific pathogen-free animal facilities on a 12 h light/dark cycle at a constant temperature (25 ± 0.5 °C) and humidity (50 ± 5%) and provided with standard chow. The experiment was carried out under the approval of the Animal Experiment Ethics Review Committee of South China Agricultural University (agreement number is SYXK 2019-0136).

### 2.3. Experimental Design

The mice were randomly divided into the control group (control, *n* = 10) and DSS group (*n* = 40). The control group was given normal saline, and the DSS group was given 3% DSS (40 kD) to induce acute UC. Seven days after modelling, the mice in the DSS group were divided into the model group and the low-dose, middle-dose and high-dose groups, with an equal number of mice in each group. The corresponding concentrations were 0.1 g/mL, 0.2 g/mL and 0.4 g/mL [9]. 0.2 mL was given by gavage every morning. The control group and the model group were given normal saline, and the treatment group was given different concentrations of *Forsythia suspensa* extract.

During the experiment, the body weight, food intake, and disease activity index (DAI) of the mice were measured every day. In addition, the amount of drinking water consumed was measured every day throughout the modelling process. The DAI is a composite of the stool status score, weight loss score and blood stool score, which were evaluated as follows: stool trait score 0: normal, 1: soft stool, 2: soft stool, 3: diarrhea, 4: severe diarrhea; weight loss score 0: no weight loss; 1: 1–5%; 2: 6–10%; 3: 10–20%; 4: over 20%; blood in the stool 0: none, 1: occult blood, 2: occult blood positive, 3: occult blood positive, 4: bloody stool. The day before the end of the experiment, the mice were fasted for 24 h. Then, the mice were euthanized, and the liver, colon and spleen were collected. The specimens were frozen in liquid nitrogen or fixed in 10% (*w*/*v*) formalin solution for storage.

### 2.4. LC–MS Analysis

LC–MS was performed in electrospray ionization (ESI) mode with a liquid chromatography instrument (U3000, Thermo Fisher, Waltham, MA, USA) combined with a mass spectrometer (QE Plus, Thermo Fisher, Waltham, MA, USA). The *Forsythia suspensa* extract was centrifuged at 12,000 rpm at 4 °C for 10 min and filtered through a 0.22 μm membrane. An ACQUITY UPLC* HSS T3 chromatographic column (1.8 µm, 2.1 × 150 mm, Waters) was used. The column temperature was 40 °C and the autosampler temperature was 8 °C. The Forsythia extract was eluted using 5 mM ammonium formate in water (A), acetonitrile (B) or 0.1% formic acid in water (C) and 0.1% formic acid in acetonitrile (D) at a flow rate of 0.25 mL/min; the injection volume was 2 μL. The gradient elution procedure was as follows: 0–1 min, 2% B/D; 1–9 min, 2–50% B/D; 9–12 min, 50–98% B/D; 12–13.5 min, 98% B/D; 13.5–14 min, 98–2% B/D; 14–20 min, 2% D- positive mode (14–17 min, 2% B-negative model). The ESI source was in the positive and negative ionization modes, the positive ion spray voltage was 3.50 kV, the negative ion spray voltage was 2.50 kV, the sheath gas was 30 arb and the auxiliary gas was 10 arb. The capillary temperature was 325 °C; a full scan was performed with a resolution of 70,000, and the range was 81–1000. The HCD was used for secondary cracking, and the collision voltage was 30 eV. Furthermore, dynamic exclusion was used to remove unnecessary secondary mass spectrometry (MS/MS) information. The identification of the chemical composition was first confirmed by the exact molecular weight (molecular weight error ≤ 30 ppm), and then the Human Metabolome Database (HMDB) (http://www.hmdb.ca (accessed 18 September 2021)), METLIN (http://metlin.scripps.edu (accessed 18 September 2021)), Massbank (http://www.massbank.jp/ (accessed 18 September 2021)), LipidMaps (http://www.lipidmaps.org (accessed 18 September 2021)), mzClound (https://www.mzcloud.org (accessed 18 September 2021)), and BioNovoGene self-built standard product database confirmed that the chemical composition was obtained.

### 2.5. Measurement of Superoxide Dismutase (SOD), Malondialdehyde (MDA) and Myeloperoxidase (MPO) Levels

Colon tissue (200 mg) was homogenized in phosphate buffered solution [PBS, 1:9 (*w*/*v*)] and centrifuged to obtain the supernatant. A kit (Nanjing Institute of Bioengineering, China) was used to measure the levels of SOD and MDA in the colon tissue. The level of MPO was evaluated with an MPO ELISA kit (Shanghai Enzyme Link Biotechnology Co., Ltd., Shanghai, China) according to the manufacturer’s instructions. MPO reflects the number of neutrophils in the colon tissue [19].

### 2.6. Cell Culture and Viability Assay

Mouse J774A.1 cells (ANWEISCI, Shanghai, China) were cultured in medium supplemented with 10% fetal bovine serum (FBS), penicillin (100 U/mL) and streptomycin (100 μg/mL). The cells were plated in a 25 cm^2^ cell culture flask and cultured in a humidified incubator at 37 °C and 5% CO_2_.

The viability of J774A.1 cells was determined by the CCK8 assay. A total of 1 × 10^5^ cells/well were plated in a 96-well plate and incubated for 24 h with different concentrations of *Forsythia suspensa* extract. Then, 10% CCK8 solution (*V/V*) was added, and the cells were incubated in an incubator for approximately 4 h. The absorbance at 570 nm wavelength was measured with a microplate reader (Multiskan MK3; Thermo Fisher, Waltham, MA, USA), and the cell growth inhibition rate was calculated.

### 2.7. Flow Cytometry

A Reactive Oxygen Species Assay Kit (Biyuntian Biotechnolog, Shanghai, China) was used to measure reactive oxygen species (ROS). Briefly, J774A.1 cells were cultured in 6-well plates, and the model group was treated with 1 ug/mL lipopolysaccharide(LPS) for 12 h, and then with 5 mM ATP for 1 h. Treatment groups were treated with 1μg/mL LPS and *Forsythia suspensa* extract (0.5 mg/mL, 1 mg/mL, and 2 mg/mL) for 12 h, and then stimulated with 5 mM ATP for 1 h. The cells were collected by pipetting, washed with PBS and centrifuged at 1000× *g* for 5 min. The cells were suspended in a medium containing 10 μmol/L DCFH-DA and then incubated at 37 °C for 30 min. The cells were then washed three times with PBS and collected. The ROS level was measured with a flow cytometer (CytoFlex; Beckman Coulter). FlowJo software (Ashland) was used to analyze the data.

### 2.8. RNA Extraction and Quantitative Real-Time PCR (qRT-PCR)

A kit (Tsingke Biotechnology Co., Ltd. (Beijing, China)) was used to extract total RNA from colon tissue or J774A.1 cells, and a TaKaRa PrimeScript RT Kit was used to transcribe RNA into cDNA. ChamQ Universal SYBR qPCR Master Mix was used for qRT–PCR following the manufacturer’s instructions. The primers were obtained from Tsingke Biotechnology Co., Ltd. and are listed in Table 1.

### 2.9. Western Blot Analysis

Tissues or cells were homogenized in RIPA buffer (Beyotime, Shanghai, China) containing phosphatase inhibitors. The protein concentration in the supernatant was measured using a BCA protein assay kit (Beyotime, Shanghai, China). The proteins were separated on an SDS–PAGE gel (Beyotime, Shanghai, China) and transferred onto the polyvinylidene fluoride(PVDF) membrane, which was blocked with 5% skimmed milk for 1.5 h. The membrane was then incubated with NLRP3 (diluted 1:1000, Cell Signaling Technology, D4D8T), Keap1(diluted 1:1000, Cell Signaling Technology, D6B12), GSDMD (diluted 1:1000, Cell Signaling Technology, E9S1X), NQO1(diluted 1:1000, Cell Signaling Technology, D6H3A), ASC (diluted 1:1000, Cell Signaling Technology, D2W8U), HO-1(diluted 1:1000, Cell Signaling Technology, E9H3A), Cleaved-Caspase-1 (diluted 1:1000, Cell Signaling Technology, E2G2I), Nrf2 (diluted 1:1000, Cell Signaling Technology, D1Z9C), IL-1β (diluted 1:1000, Cell Signaling Technology,3A6) and β-actin (diluted 1:1000, Cell Signaling Technology,13E5) antibodies for more than 16 h, then were rinsed three times with tris buffered saline tween (TBST) and incubated with horseradish peroxidase-conjugated secondary antibodies (diluted 1:5000) for 1 h. Enhanced chemiluminescence (ECL) reagent (Beyotime, Shanghai, China) was used to visualize the protein bands. The results were analyzed by Image-Pro Plus software (Image V1.8.0).

### 2.10. Isolation of Nuclear Proteins

Cytoplasmic protein extraction reagent A containing phenylmethylsulfonyl fluoride (PMSF) was added to the cell or tissue homogenate, and the samples were placed in an ice bath for 10 min after shaking. Then, cytoplasmic protein extraction reagent B was added, and the samples were placed in an ice bath for 1 min after shaking. The samples were centrifuged at 14,000× *g* for 5 min, and the supernatant was collected. After the supernatant was aspirated completely, nuclear protein extraction reagent containing PMSF was added to the remaining precipitate. The samples were placed in an ice bath with shaking for 30 min and centrifuged at 14,000× *g* for 10 min, and the supernatant was collected as nuclear protein.

### 2.11. Lactate Dehydrogenase (LDH) Activity Assay

An LDH Release Assay Kit (Beyotime, Shanghai, China) was used to measure the activity of LDH released during cytotoxicity by colorimetry. Briefly, 1 h before the end of the experiment, 10% LDH release reagent was added to the wells to measure the maximum enzyme activity of the samples. After adding the LDH release reagent, the samples were mixed well, and then the cells were incubated in a cell culture incubator. Finally, the absorbance was measured at 490 nm.

### 2.12. Sample Preparation and Metabolite Detection

Serum samples were placed at 4 °C, and then 50 µL of each sample was placed in a 2 mL centrifuge tube; 400 µL of methanol was added, and the samples were shaken for 60 s and mixed thoroughly. The samples were centrifuged at 12,000 rpm and 4 °C for 10 min, and then the supernatant was transferred to a new 2 mL centrifuge tube and concentrated in vacuo to dryness. The samples were diluted with 150 µL 2-chlorobenzalanine (4 ppm) in 80% methanol, and the supernatant was filtered through a 0.22 µm membrane to obtain the prepared samples for liquid chromatograph-mass spectrometer (LC–MS). A total of 20 µL of each sample to be tested was mixed into a quality control (QC) sample, and the remaining sample to be tested was used for LC–MS. Finally, analysis was carried out by LC–MS.

### 2.13. Metabolite Data Processing and Analysis

R language (R 3.3.2) was used to perform multivariate analysis of metabolite parameters, including the mass-to-nucleus ratio, peak area and retention time, to identify specific metabolites in the dataset. The exact molecular weight of the identified metabolites (molecular weight error <30 ppm) was first confirmed, and then the fragment information obtained in MS/MS mode was verified with the HumanMetabolome Database (HMDB) (http://www.hmdb.ca (accessed 18 September 2021)), Metlin (http://metlin.scripps.edu (accessed 18 September 2021)), Massbank (http://www.massbank.jp/ (accessed 18 September 2021)), LipidMaps (http://www.lipidmaps.org (accessed 18 September 2021)), mzclound (https://www.mzcloud.org (accessed 18 September 2021)) and Pano Mick’s self-built standard product database. The Kyoto Encyclopedia of Genes and Genomes (KEGG) (http://www.kegg.jp/ (accessed 18 September 2021)) database was used to map the metabolomics pathway network. We used Metaboanalyst (www.metaboanalyst.ca (accessed 18 September 2021)) to conduct pathway enrichment analysis to screen related metabonomic pathways related to the identified metabolites.

For metabolomics analysis, 6 biological replicates were included for each group. Orthogonal projection of latent structure discriminant analysis (OPLS-DA) was used to filter the metabolites, effectively reducing the complexity and enhancing the interpretation ability of the model without reducing its predictive ability to maximize the difference between groups. Multivariate statistical scores were used to determine whether there were significant differences in metabolite levels (*p* ≤ 0.05) between the control group, model group, low-dose group (L-dose), medium-dose group (M-dose) and high-dose groups (H-dose). Metabolites with a VIP ≥ 1 and *p* ≤ 0.05 were identified as potential biomarkers.

### 2.14. Histological Analysis

Colon tissue was stained with hematoxylin and eosin (H&E), and light microscopy was used to observe tissue damage and inflammation. Histological evaluation followed the scoring system previously described [6].

### 2.15. Statistical Analysis

The data were analysed using SPSS 20.0 software (IBM, Armonk, NY, USA). GraphPad Prism 5.0 software (GraphPad; San Diego, CA, USA) was used to generate graphs. The experimental results are shown as the mean ± standard error of mean (SEM) of at least three independent experiments. The data were analyzed by one-way analysis of variance (ANOVA) and Fisher’s least significant difference (LSD) test. *p* < 0.05 was considered significant.

## 3. Results

### 3.1. Identification of the Chemical Constituents of Forsythia suspensa Extract

Previous studies have found that *Forsythia suspensa* polyphenol extract has high antioxidant activity and good free radical scavenging ability [20,21]. Phytochemical analysis of *Forsythia suspensa* extract was performed by LC–MS (Table 2). Seven polyphenol metabolites, including homovanillic acid, hydroquinone, isoproterenol, norepinephrine, p-synephrine, sinapyl alcohol and tyrosol, were preliminary identified in *Forsythia suspensa* extract.

### 3.2. Forsythia suspensa Extract Alleviates DSS-Induced Colitis

**Figure 1:**
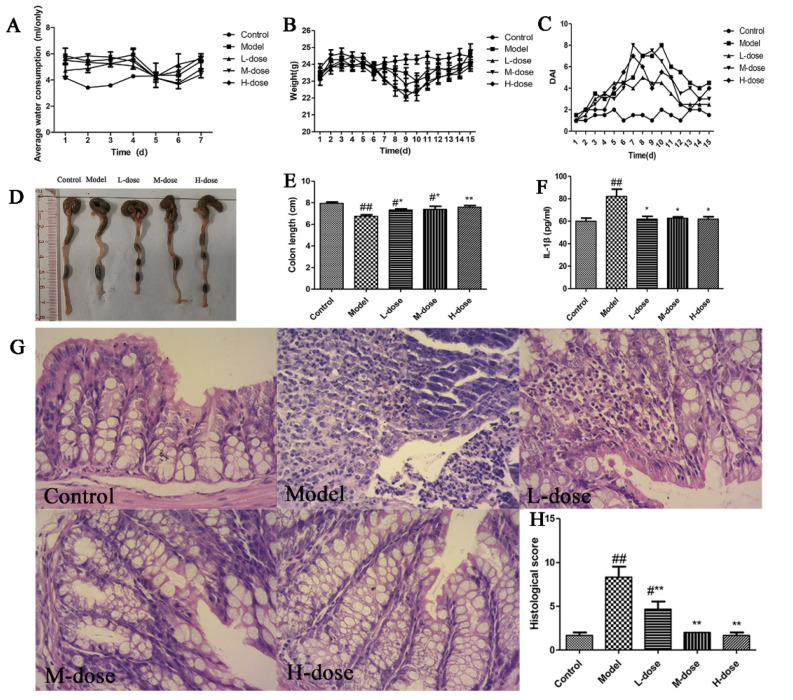
*Forsythia suspensa* extract alleviates the symptoms of UC in mice. (**A**) Average water consumption during modelling. (**B**) Average weight of the mice (*n* = 10). (**C**) DAI score. (**D**) Representative pictures of the colon. (**E**) Average colon length (*n* = 10). (**F**) Measurement of the level of IL-1β in the serum with an ELISA kit. (**G**) Pathological examination of mouse colon tissue (×200). (**H**) Histological score of the colon. Data represent at least three independent experiments. The results are shown as the mean ± SEM. ^#^ *p* < 0.05, and ^##^ *p* < 0.01 compared with the control group; * *p* < 0.05, and ** *p* < 0.01 compared with the model group; #** means # and **.

To verify whether *Forsythia suspensa* extract can ameliorate colon injury and inflammation, the effect of the extract of *Forsythia suspensa* on mice with 3% DSS-induced UC was assessed. First, it was found that there was little difference in the trend in water consumption during the 7 days of modelling between the groups (Figure 1A). In the model building model, the weight of the mice in the DSS group decreased. After drug administration, the weight of the mice in the treatment group increased (Figure 1B), and the DAI score decreased (Figure 1C). As shown in Figure 1D,E, *Forsythia suspensa* extract alleviated colon shortening. In addition, the level of IL-1β in the serum was measured, and the results showed that *Forsythia suspense* extract significantly reduced the levels of this inflammatory factor (Figure 1F). Histopathological analysis revealed that the control group, M-dose group and H-dose group exhibited normal tissue structure without obvious pathological changes; however, the model group and L-dose group showed intestinal villi degeneration, necrosis, proliferation and infiltration of a large number of inflammatory cells (Figure 1G). 

### 3.3. Effects of Forsythia suspensa Extract on Oxidative Stress and MPO Levels in Mice

**Figure 2:**
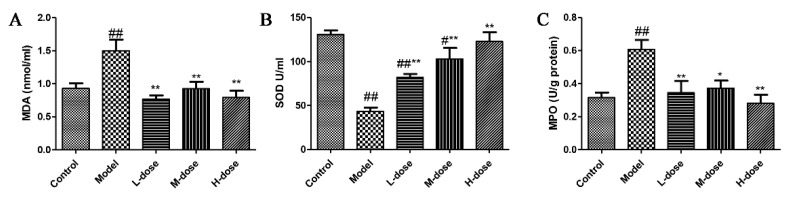
The effect of *Forsythia suspensa* extract on the levels of MDA, SOD and MPO in mice. (**A**) MDA level in serum. (**B**) SOD level in serum. (**C**) MPO activity in colon tissue. Data represent at least three independent experiments. The results are shown as the mean ± SEM. ^#^ *p* < 0.05, and ^##^ *p* < 0.01 compared with the control group; * *p* < 0.05, and ** *p* < 0.01 compared with the model group; #** means # and **; ##** means ## and **.

To evaluate the effect of *Forsythia suspensa* extract on colonic mucosal damage and neutrophil infiltration, the levels of MDA, SOD and MPO were tested. As shown in Figure 2A,B, *Forsythia suspensa* extract significantly reduced the level of MDA and significantly increased the level of SOD. These results indicate that *Forsythia suspensa* extract can alleviate the level of DSS-induced oxidative stress in mice. In addition, *Forsythia suspensa* extract significantly reduced the MPO activity of colon tissue (Figure 2C).

### 3.4. Antioxidant Effect of Forsythia suspensa Extract

**Figure 3:**
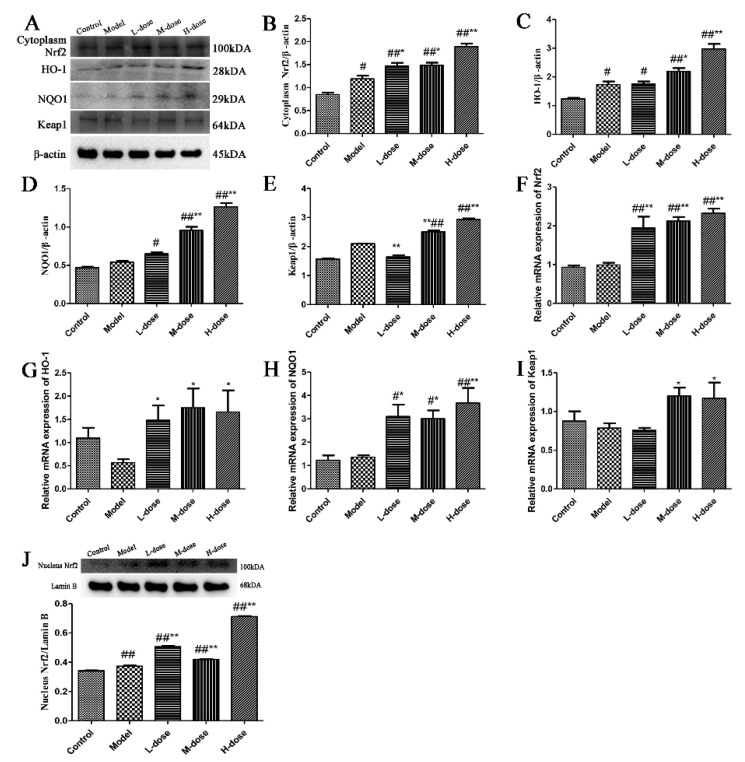
*Forsythia suspensa* extract enhances the antioxidant activity in mouse colon tissue. (**A**–**E**) Western blot analysis of cytoplasm for Nrf2, HO-1, NQO1 and Keap1. (**F**–**I**) qRT–PCR detection of *Nrf2*, *HO-1*, *NQO1* and *Keap1* gene expression levels. (**J**) Western blot analysis of the level of nuclear Nrf2. The results are shown as the mean ± SEM. ^#^ *p* < 0.05, and ^##^ *p* < 0.01 compared with the control group; * *p* < 0.05, and ** *p* < 0.01 compared with the model group; #** means # and **; ##** means ## and **.

Studies have found that forsythoside A, isoforsythoside A, phillyrin, forsythialan A, phillygenin and polysaccharides, which are components of *Forsythia suspensa* that have strong antioxidant effects [10,22]. To determine the antioxidant effect of *Forsythia suspensa* extract, the expression levels of Nrf2, HO-1, NQO1 and Keap1 in colon tissue were further analyzed. Western blotting results showed that the levels of HO-1, NQO1 and Keap1 in the treatment group were significantly increased compared with those in the control group (Figure 3A,C–E) and that the levels of Nrf2 in the cytoplasm and nucleus were also significantly increased (Figure 3B,J). To further evaluate the effects of *Forsythia suspensa* extract, the levels of these genes in colon tissue were also examined by qRT–PCR. The results showed that *Forsythia suspense* extract promoted the expression of *Nrf2*, *HO-1*, *NQO1* and *Keap1* (Figure 3F–I). These results show that *Forsythia suspense* extract enhances the antioxidant activity in mouse colon tissue.

### 3.5. Forsythia suspensa Extract Inhibits the Occurrence of Pyroptosis

**Figure 4:**
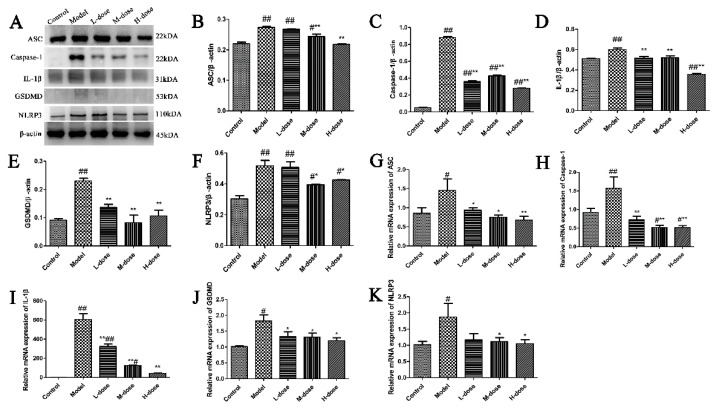
*Forsythia suspensa* extract may alleviate colitis by inhibiting pyroptosis. (**A**–**F**) Western blot analysis of ASC, Caspase-1, IL-1β, GSDMD and NLRP3. (**G**–**K**) qRT–PCR detection of *ASC*, *Caspase-1*, *IL-1β*, *GSDMD* and *NLRP3* gene expression levels. The results are shown as the mean ± SEM. ^#^ *p* < 0.05, and ^##^ *p* < 0.01 compared with the control group; * *p* < 0.05, and ** *p* < 0.01 com-pared with the model group; #** means # and **; ##** means ## and **.

Pyroptosis is a type of programmed cell death that is involved in experimental colitis in mice [23,24]. Therefore, to explore whether *Forsythia suspensa* extract exerts anti-inflammatory effects by inhibiting cell pyroptosis, we measured the expression of pyroptosis-related genes. Western blotting showed that ASC, Caspase-1, IL-1β, GSDMD and NLRP3 expression in the model group was increased and that the expression levels of these proteins in the *Forsythia suspensa* extract-treated group were significantly reduced (Figure 4A–E). In addition, the qRT–PCR results showed that the expression of the *ASC*, *Caspase-1*, *IL-1β*, *GSDMD* and *NLRP3* genes in the *Forsythia suspensa* extract-treated group was significantly reduced compared with that in the model group (Figure 4G–K). These experimental results indicate that *Forsythia suspensa* extract may alleviate colitis by inhibiting pyroptosis.

### 3.6. Effect of Forsythia suspensa Extract on Cell Viability

We primarily used the CCK-8 method to assess cell viability. As shown in Figure S1, the cell survival rate was inhibited by more than 50% after treatment with 8 mg/mL *Forsythia suspensa* extract. Therefore, 0.5 mg/mL, 1 mg/mL and 2 mg/mL *Forsythia suspensa* extract were used in the next in vitro experiments.

### 3.7. The effect of Forsythia suspensa Extract on a Pyroptosis Model In Vitro

**Figure 5:**
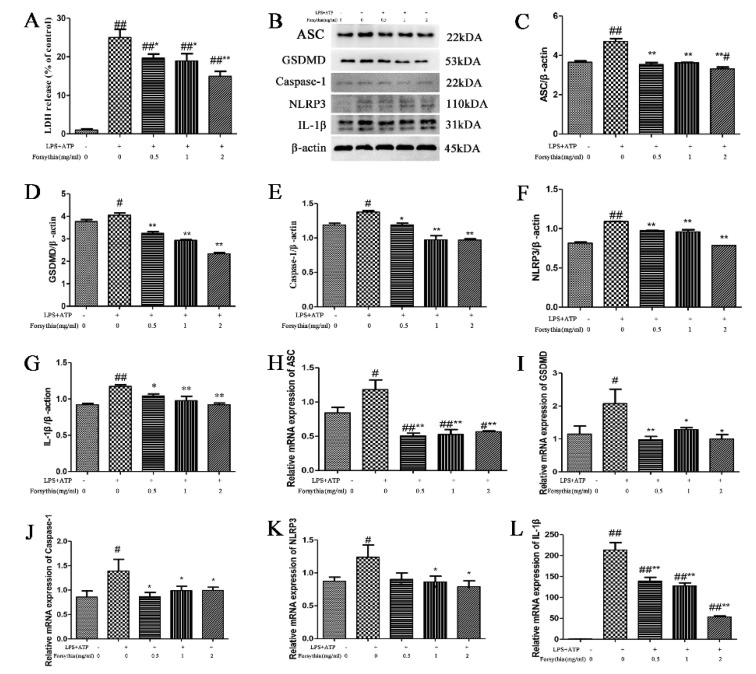
*Forsythia suspensa* extract can reduce the protein and gene expression of pyroptosis in J774A.1 cells. (**A**) J774A.1 cells were first treated with 1 μg/mL LPS and different concentrations of *Forsythia suspensa* extract for 12 h, and then stimulated with 5 mM ATP for 1 h. The kit detects the level of LDH release. (**B**–**G**) Western blot analysis of ASC, Caspase-1, IL-1β, GSDMD and NLRP3. (**H**–**L**) qRT–PCR detection of *ASC*, *Caspase-1*, *IL-1β*, *GSDMD* and *NLRP3* gene expression levels. Data represent at least three independent experiments. The results are shown as the mean ± SEM. ^#^ *p* < 0.05, and ^##^ *p* < 0.01 compared with the control group; * *p* < 0.05, and ** *p* < 0.01 compared with the model group; #** means # and **; ##** means ## and **.

First, a kit was used to measure the amount of LDH released by J774A.1 cells. The results indicated that *Forsythia suspensa* extract reduced LDH levels in a dose-dependent manner (Figure 5A). Compared with that in the control group, the expression of pyroptosis-related proteins in the model group was increased, proving that an in vitro pyroptosis model was successfully established. Western blot analysis revealed that *Forsythia suspensa* extract dose-dependently reduced the protein levels of ASC, Caspase-1, IL-1β, GSDMD and NLRP3 (Figure 5B–G). Furthermore, the qRT–PCR results showed that *Forsythia suspensa* extract reduced the gene expression of the *ASC*, *Caspase-1*, *IL-1β*, *GSDMD* and *NLRP3* genes (Figure 5H–L). The results showed that *Forsythia suspensa* extract can reduce the protein and gene expression of pyroptosis in J774A.1 cells.

### 3.8. Forsythia suspensa Extract Inhibits the Oxidative Stress Response

**Figure 6:**
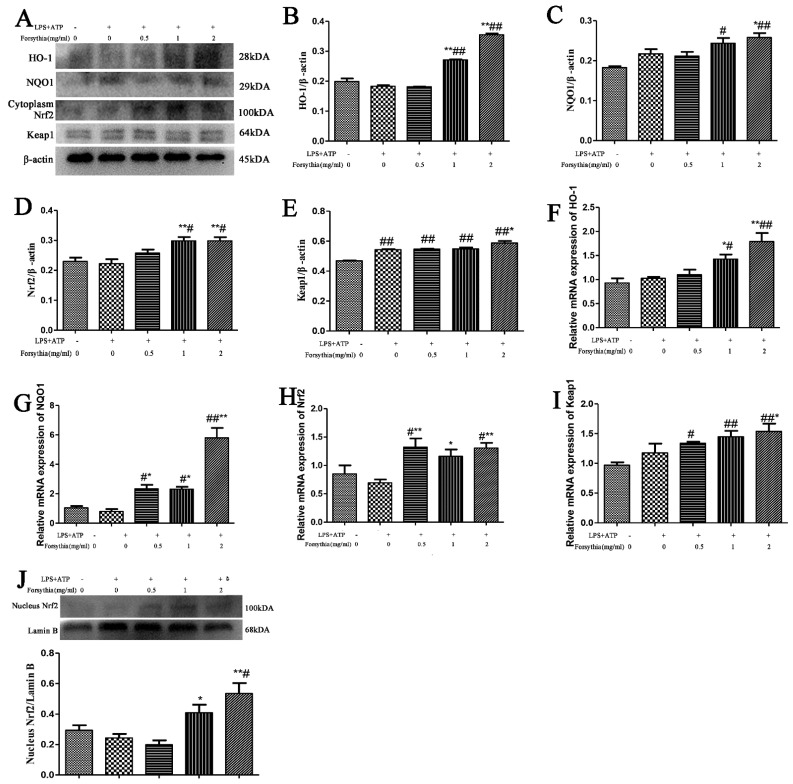
*Forsythia suspensa* extract enhances the antioxidant activity J774A.1 cells. (**A**–**E**) Western blot analysis of HO-1, NQO1, and cytoplasm for Nrf2 and Keap1. (**F**–**I**) qRT–PCR detection of *HO-1*, *NQO1*, *Nrf2* and *Keap1* gene expression levels. (**J**) Western blot analysis of the level of nuclear Nrf2. The results are shown as the mean ± SEM. ^#^ *p* < 0.05, and ^##^ *p* < 0.01 compared with the control group; * *p* < 0.05, and ** *p* < 0.01 compared with the model group; #** means # and **; ##** means ## and **.

To confirm whether *Forsythia suspensa* extract can improve the antioxidant activity in J774A.1 cells, we used a cell pyroptosis model. Western Blotting results showed that, compared with those in the control group, the levels of HO-1, NQO1, Keap1 and cytoplasmic Nrf2 in the treatment group were significantly increased (Figure 6A–E). Further research revealed that the level of Nrf2 in the nucleus was also significantly increased (Figure 6J). In addition, qRT–PCR was used to measure the expression levels of these genes in cells. The results showed that *Forsythia suspensa* extract promoted the gene expression of *HO-1*, *NQO1*, *Nrf2* and *Keap1* (Figure 6F–I). These results indicate that *Forsythia suspensa* extract enhances the antioxidant activity in J774A.1 cells.

### 3.9. Forsythia suspensa Extract Reduces ROS Levels in J774A.1 Cells

**Figure 7:**
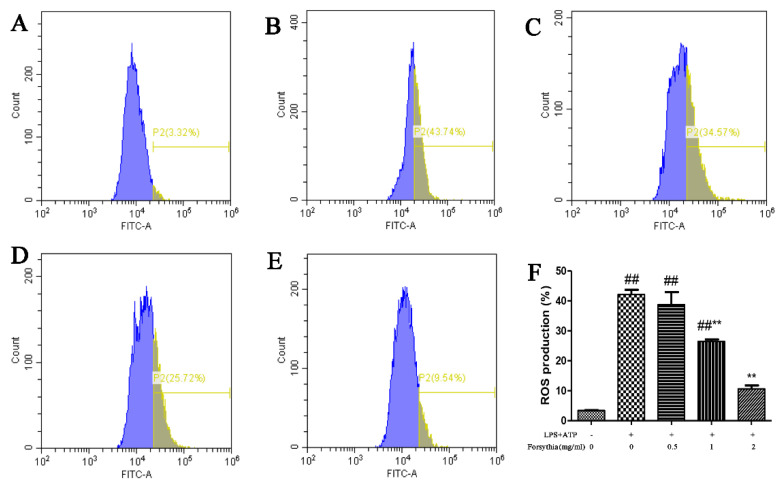
*Forsythia suspensa* extract significantly reduces the level of ROS in J774A.1 cells. (**A**) Control group. (**B**) Model group. (**C**) 0.5 mg/mL *Forsythia suspensa* extract. (**D**) 1 mg/mL *Forsythia suspensa* extract. (**E**) 2 mg/mL *Forsythia suspensa* extract; (**F**) Statistical analysis of ROS levels in J774A.1 cells. Data represent at least three independent experiments. The results are shown as the mean ± SEM. ^##^ *p* < 0.01 compared with the control group;** *p* < 0.01 compared with the model group; #** means # and **; ##** means ## and **.

Studies have reported that excessive ROS are an important trigger of apoptosis, autophagy and pyroptosis [25]. Therefore, in order to study whether *Forsythia suspensa* extract can reduce ROS levels in J774A.1 cells, we used DCFH-DA as a fluorescent probe and flow cytometry to measure intracellular ROS levels. The results showed that *Forsythia suspensa* extract significantly reduced the level of ROS in J774A.1 cells (Figure 7A–F).

### 3.10. Forsythia suspensa Extract Inhibits Pyroptosis via Activation of the NLRP3 Inflammasome

**Figure 8:**
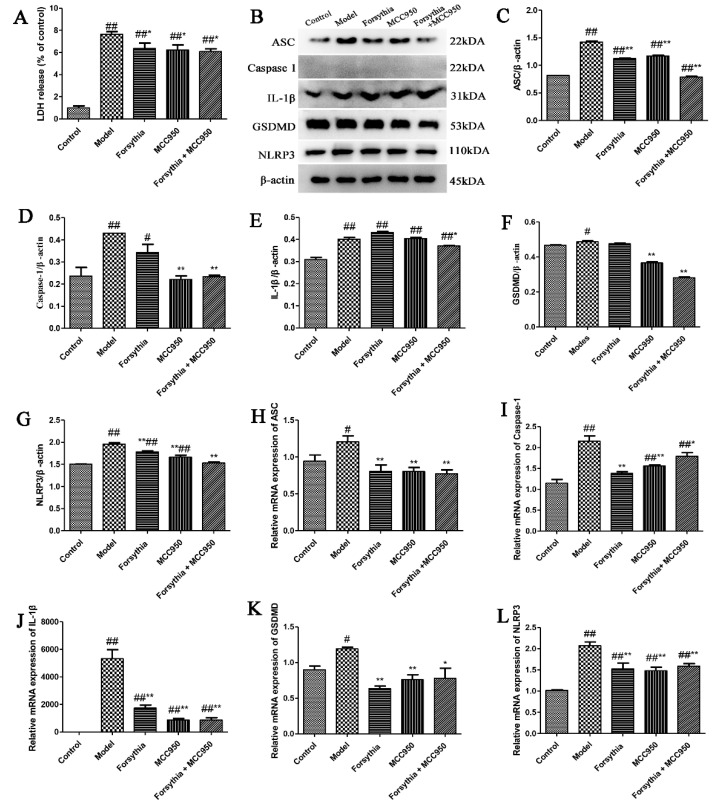
*Forsythia suspensa* extract may block the occurrence of pyroptosis by inhibiting the activation of NLRP3. (**A**) LDH levels in J774A.1 cells. (**B**–**G**) Western blot analysis of ASC, Caspase-1, IL-1β, GSDMD and NLRP3. (**H**–**L**) qRT–PCR detection of *ASC*, *Caspase-1*, *IL-1β*, *GSDMD* and *NLRP3* gene expression levels. Data represent at least three independent experiments. The results are shown as the mean ± SEM.^#^ *p* < 0.05, and ^##^ *p* < 0.01 compared with the control group; * *p* < 0.05, and ** *p* < 0.01 compared with the model group; #** means # and **; ##** means ## and **.

To determine the role of the NLRP3 inflammasome in the *Forsythia suspensa* extract-mediated pyroptosis, we used the inhibitor MCC950 (an NLRP3 inhibitor). First, the release of LDH in J774A.1 cells was assessed, and the results showed that *Forsythia suspensa* extract significantly reduced the amount of LDH released by J774A.1 cells (Figure 8A). Western blot analysis showed that *Forsythia suspensa* extract reduced the protein levels of ASC, Caspase-1, IL-1β, GSDMD and NLRP3, and that the inhibitor MCC950 also decreased the level of pyroptosis associated protein (Figure 8B–G). In addition, qRT–PCR showed that *Forsythia suspensa* extract reduced the gene expression of *ASC*, *Caspase-1*, *IL-1β*, *GSDMD* and *NLRP3* genes, and that the inhibitor MCC950 showed a similar effect (Figure 8H–L). The results indicate that *Forsythia suspensa* extract may block the occurrence of pyroptosis by inhibiting the activation of NLRP3.

### 3.11. The Nrf2 Signalling Pathway Plays a Role in the Process by Which Forsythia suspensa Extract Inhibits Pyroptosis

**Figure 9:**
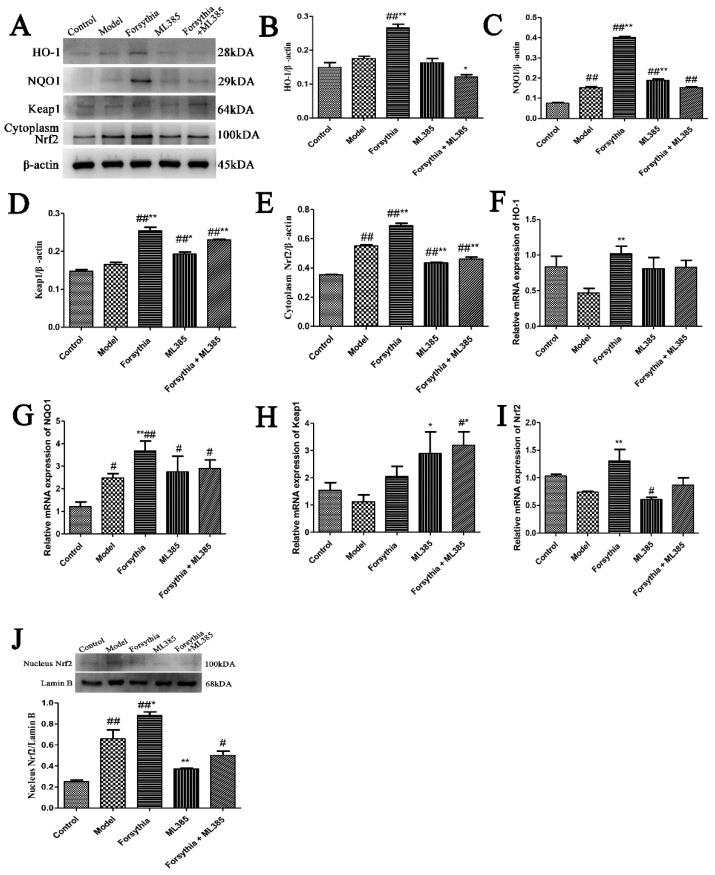
The inhibitor ML385 reverses the antioxidant effect of *Forsythia suspensa* extract. (**A**–**E**) Western blot analysis of HO-1, NQO1, Keap1 and cytoplasm for Nrf2. (**F**–**I**) qRT–PCR detection of *HO-1*, *NQO1*, *Keap1* and *Nrf2* gene expression levels. (**J**) Western blot detects the level of nuclear Nrf2. The results are shown as the mean ± SEM. ^#^ *p* < 0.05, and ^##^ *p* < 0.01 compared with the control group; * *p* < 0.05, and ** *p* < 0.01 compared with the model group; #** means # and **; ##** means ## and **.

To explore the potential role of Nrf2 in colonic inflammation, we used the inhibitor ML385 to inhibit Nrf2 in J774A.1 cells. Western blotting showed that, compared with the control, *Forsythia suspensa* extract significantly increased HO-1, NQO1 and Keap1 levels, and significantly increased Nrf2 levels in the cytoplasm and nucleus, while ML385 reversed the effect of *Forsythia suspensa* extract (Figure 9A–J). The results of qRT–PCR showed that *Forsythia suspensa* extract increased antioxidant activity and that ML385 reduced the gene expression of the *HO-1*, *NQO1*, *Keap1* and *Nrf2* genes (Figure 9F–I). These results indicate that ML385 reverses the antioxidant effect of the *Forsythia suspensa* extract.

**Figure 10:**
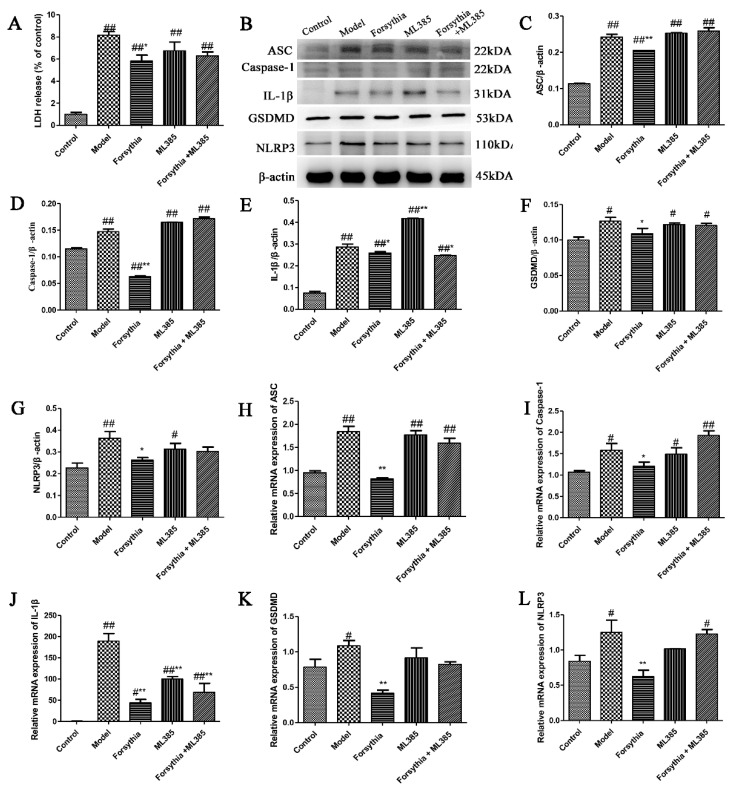
The effect of *Forsythia suspensa* extract in inhibiting pyroptosis may be related to its antioxidant effect. (**A**) LDH levels in J774A.1 cells. (**B**–**G**) Western blot analysis of ASC, Caspase-1, IL-1β, GSDMD and NLRP3. (**H**–**L**) qRT–PCR detection of *ASC*, *Caspase-1*, *IL-1β*, *GSDMD* and *NLRP3* gene expression levels. Data represent at least three independent experiments. The results are shown as the mean ± SEM. ^#^ *p* < 0.05, and ^##^ *p* < 0.01 compared with the control group; * *p* < 0.05, and ** *p* < 0.01 compared with the model group; ##* means ## and *; ##** means ## and **.

The results showed that the inhibitor ML385 could reverse the inhibitory effect of *Forsythia suspensa* extract on LDH release (Figure 10A). Western blot analysis showed that *Forsythia suspensa* extract reduced the protein levels of ASC, Caspase-1, IL-1β, GSDMD and NLRP3 and that ML385 reversed this effect (Figure 10B–G). In addition, qRT–PCR revealed that *Forsythia suspensa* extract inhibited the expression of pyroptosis-related genes and that ML385 prevented the inhibition of pyroptosis by *Forsythia suspensa* extract (Figure 10F–L). The results indicate that the effect of *Forsythia suspensa* extract in terms of inhibitory pyroptosis may be related to its antioxidant effect.

### 3.12. Multivariate Statistical Analysis of Metabolites

**Figure 11:**
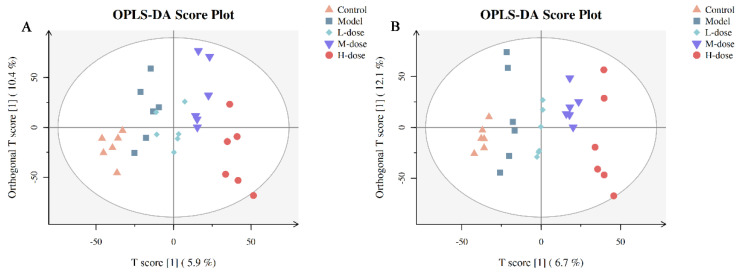
The OPLS-DA scores of the control, model, L-dose, M-dose and H-dose groups are based on the serum metabolite curve in positive and negative ion modes. (**A**) Positive ion mode; (**B**) Negative ion mode.

A VIP of a variable is >1, which indicates that a variable is important, and this was used as one of the screening conditions for potential biomarkers. A *p* value ≤ 0.05 was further used to screen differentially expressed metabolites, which were regarded as potential biomarkers. OPLS-DA was used to identify metabolites that were expressed at different levels in the different groups. Compared with the unsupervised PCA, OPLS-DA focuses more on the actual category discrimination changes to maximize the viewing of differences between groups. Figure 11 shows the OPLS-DA scores of the five groups. There was a clear separation among the control group, model group, L-dose, M-dose and H-dose groups (Figure 10A,B). The H-dose group was farthest from the model group, followed by the M-dose group and control group, and the model group and the L-dose group were close together. OPLS-DA score analysis showed that *Forsythia suspensa* extract alleviated metabolic dysfunction in mice with DSS-induced colitis.

### 3.13. Metabolite Identification

**Figure 12:**
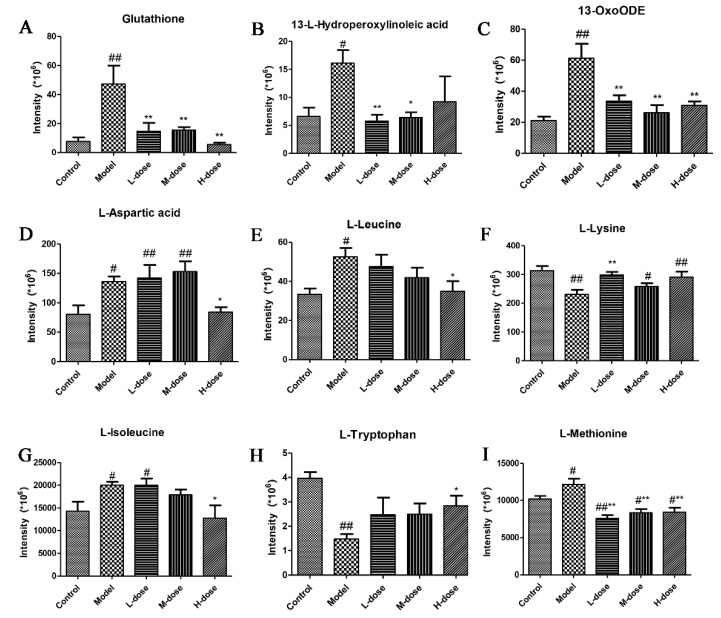
Serum levels of nine metabolites. (**A**) Glutathione, (**B**) 13-L-hydroperoxylinoleic acid, (**C**) 13-oxoODE, (**D**) L-aspartic acid, (**E**) L-leucine, (**F**) L-lysine, (**G**) L-isoleucine, (**H**) L-tryptophan and (**I**) L-methionine. Data represent at least three independent experiments. The results are shown as the mean ± SEM. ^#^ *p* < 0.05, and ^##^ *p* < 0.01 compared with the control group; * *p* < 0.05, and ** *p* < 0.01 compared with the model group; #** means # and **; ##** means ## and **.

The metabolites were first confirmed based on the exact molecular weight (molecular weight error is <30 ppm) and then based on the MS/MS fragmentation model for Metlin (http://metlin.scripps.edu (accessed 18 September 2021)), MoNA (https://mona.fiehnlab.ucdavis.edu (accessed 18 September 2021)), and Panomik’s self-built standard database. These were used to confirm the annotations of the metabolites. According to the above method, 77 differential metabolic signatures were screened (Table S1). Nine ions were identified, and the results are shown in Figure 12. Analysis of the results showed that the levels of glutathione, 13-L-hydroperoxylinoleic acid, 13-OxoODE, L-aspartic acid, L-leucine, L-isoleucine and L-methionine were increased, and the levels of L-lysine and L-tryptophan were decreased in the model group (Figure 12A–I). However, the levels of these metabolites were reversed after *Forsythia suspensa* extract treatment.

### 3.14. Analysis of Potential Metabolic Pathways

**Figure 13:**
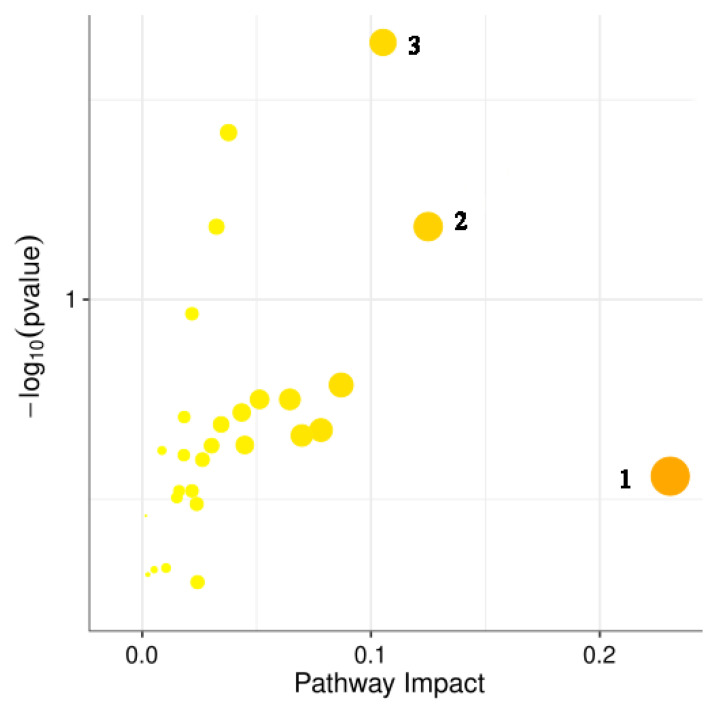
Metabolic pathway analysis based on the potential biomarkers. 1, glutathione metabolism; 2, aminoacyl-tRNA biosynthesis; 3, linoleic acid metabolism.

To further determine the potential metabolic pathways affected by *Forsythia suspensa* extract, these potential biomarkers were introduced into MetaboAnalyst. We performed enrichment analysis of the nine differentially expressed metabolites and identified potential metabolic pathways associated with the metabolites. The results showed that glutathione metabolism, aminoacyl-tRNA biosynthesis, and linoleic acid metabolism are potentially targeted by *Forsythia suspensa* extract in the treatment of UC in mice (Figure 13).

## 4. Discussion

UC is a global and challenging disease with global prevalence, in which the body’s immune system is overactivated. It can occur at all ages and cause lifelong morbidity or even death [26,27]. Due to a high drug resistance rate, therapies for UC are not clinically satisfactory, and alternative treatment strategies are urgently needed. Therefore, scientists are paying attention to natural medicines with fewer side effects [28]. In this study, we confirmed that *Forsythia suspensa* extract can ameliorate DSS-induced UC in mice. *Forsythia suspensa* extract increased body weight and colon length in mice, and decreased DAI, indicating that *Forsythia suspensa* extract has the potential to treat UC. Our research shows that *Forsythia suspensa* extract inhibits pyroptosis in the context of UC and enhances antioxidation activity to exert an anti-inflammatory effect.

Phytochemical studies of *Forsythia suspensa* have shown that the main components of *Forsythia suspensa* fruit are triterpenoids, flavonoids, lignans and phenylethanol glycosides. Studies have clarified that phenolic compounds of *Forsythia suspensa*, including phenethyl alcohol glycosides, lignans and flavonoids, are the main contributors to the various biological effects of *Forsythia suspensa* [29]. Water extract of *Forsythia suspensa* is commonly used to treat inflammatory diseases [30]. Studies have shown that *Forsythia suspensa* extract has the potential to treat UC [9]. In addition, studies have found that *Forsythia suspensa* extract reduces the potential for inflammatory liver injury in rats by promoting antioxidants [12].

As expected, *Forsythia suspensa* extract has a therapeutic effect against DSS-induced UC in mice. In this study, DSS-treated mice showed sustained weight loss, elevated histological scores, a shortened colon and inflammatory cell infiltration. However, *Forsythia suspensa* extract treatment alleviated these manifestations. In addition, *Forsythia suspensa* extract reduced oxidative stress and MPO activity to alleviate colitis. These experimental results indicate that *Forsythia suspensa* extract may be a potential drug for the treatment of UC.

First, we studied the potential mechanism by which *Forsythia suspensa* extract relieves colitis in mice. It has been reported that *Forsythia suspensa* extract is a potential dietary antioxidant and exerts a protective effect against oxidative stress caused by diquat [10]. The protective effect of sarsasapogenin against TNBS-induced UC in rats has been reported to be related to oxidative stress [31]. Our research showed that *Forsythia suspensa* extract can increase the protein and gene expression levels of Keap1, HO-1, Nrf2 and NQO1 in mice with colitis. In addition, our study found that *Forsythia suspensa* extract can inhibit the protein and gene expression levels of NLRP3, ASC, Caspase-1 and GSDMD in colitis mice. In conclusion, our research shows that *Forsythia suspensa* extract can exert anti-inflammatory effects by enhancing antioxidant activity and inhibiting pyroptosis.

We proved in vitro that the effect of *Forsythia suspensa* extract in inhibiting pyroptosis in vitro is related to antioxidation. *Forsythia suspensa* may exert an anti-inflammatory effect by inhibiting the activation of JAK-STAT and p38 MAPK pathways in macrophages and reducing the production of ROS [32]. Studies have found that forsythiaside A inhibits the LPS-induced inflammatory response in BV2 microglia by promoting the Nrf2/HO-1 signalling pathway [33]. The anti-inflammatory effects of koreanaside A on LPS-induced macrophages and DSS-induced colitis are related to inflammatory regulation of the AP-1, NF-κB and STAT1/3 signalling pathways [34]. In addition, multiple studies have shown that the anti-inflammatory effects of drugs are related to their antioxidant properties [27,35,36]. We found that *Forsythia suspensa* extract enhanced cellular antioxidation activity and inhibited pyroptosis. Further research showed that after NLRP3 expression was inhibited by MCC950, *Forsythia suspensa* extract could no longer inhibit pyroptosis, showing that NLRP3 is necessary for pyroptosis. Silencing of NLRP3 reversed the anti-inflammatory effect of *Forsythia suspensa* extract, which indicates that inhibition of pyroptosis is one of the main mechanisms by which *Forsythia suspensa* extract relieves cell death in colitis. In addition, after inhibiting the expression of Nrf2 with ML385, the antioxidant effect of *Forsythia suspensa* extract was reduced, and the ability of *Forsythia suspensa* extract to inhibit pyroptosis was reduced. These results indicate that *Forsythia suspensa* extract may alleviate DSS-induced UC damage through the Nrf2-NLRP3 pathway. Further analysis showed that the inhibitory effect of *Forsythia suspensa* extract on pyroptosis may be related to its antioxidant effect.

Metabolomics has been widely used to determine metabolite profiles, mainly to explore the pathophysiology of diseases and to predict potential biomarkers and drug targets [37]. The results showed that *Forsythia suspensa* extract reversed dysfunction in the colon caused by alterations in glutathione metabolism, aminoacyl tRNA biosynthesis and linoleic acid metabolism. Amino acids and their derivatives play an important role in life activities and are involved in many biosynthesis processes and metabolic processes in the body. Glutathione is produced by glycine, glutamic acid and cysteine and is an important antioxidant in vivo. Previous studies have found that cysteine and glutathione can protect against damage resulting from oxidant-induced colitis in rats [38]. Studies have found that the aminoyl TRNA biosynthesis pathway is promoted in gastric cancer and plays an important role in the progression of the aminoyl-tRNA biosynthesis pathway in gastric cancer [39]. Lipids are important substances for energy storage and energy supply in vivo, and excessively oxidized lipids in the intestine cause intestinal epithelial tissue damage [40,41]. Studies have found that berberine inhibits the expression of metabolites related to linoleic acid metabolism and sheath lipid metabolism in colitis, possibly due to alterations in the metabolism of intestinal microorganisms adjusting their metabolism and reductions in the oxidation reaction in the intestine and protecting the intestinal epithelium [42].

## 5. Conclusions

In conclusion, our research results indicate that *Forsythia suspensa* extract may mediate pyroptosis through the Nrf-NLRP3 pathway to exert anti-inflammatory effects. In addition, *Forsythia suspensa* extract reverses metabolic dysfunction in the colon caused by alterations in glutathione metabolism, aminoacyl tRNA biosynthesis and linoleic acid metabolism. These results indicate that *Forsythia suspensa* extract may be a potential drug for the treatment of UC.

## Figures and Tables

**Table 1:** Sequences of primers used for qRT–PCR.

Gene		Gene Sequence	Accession Number
*β-actin*	Forward primer	TGCTGTCCCTGTATGCCTCTG	NM_007393
	Reverse primer	CTGTAGCCACGCTCGGTCA	
*NLRP3*	Forward primer	TGAACAGAGCCCCTGTAGGTAG	XM_036156549
	Reverse primer	TTGTTCTTTATCCACTGCCGAG	
*GSDMD*	Forward primer	GAAAGATTTTACAGGACCAGCC	XM_006521343
	Reverse primer	CTTGACAATAGGAACAGGGAGG	
*Keap1*	Forward primer	GCCCGGGAGTATATCTACATGC	NM_001110307
	Reverse primer	CATCCGCCACTCATTCCTCT	
*Caspase-1*	Forward primer	TTCAAAAATTGCATCCGTTAAG	NM_009807
	Reverse primer	TTGAAAGACAAGCCCAAGGTG	
*ASC*	Forward primer	TCTTGTCTTGGCTGGTGGTCT	NM_023258
	Reverse primer	ATCTGGAGTCGTATGGCTTGG	
*Nrf2*	Forward primer	CTTCCATTTACGGAGACCCAC	NM_010902
	Reverse primer	CATTGGGATTCACGCATAGGA	
*HO-1*	Forward primer	GCTGGTGATGGCTTCCTTGT	NM_010442
	Reverse primer	GCATAGACTGGGTTCTGCTTGTT	
*NQO1*	Forward primer	AGGACGCCTGAGCCCAGATA	XM_036153810
	Reverse primer	CTGGAAAGGACCGTTGTCGTAC	
*IL-1β*	Forward primer	AGGCAGGCAGTATCACTCATTG	XM_006498795
	Reverse primer	CGTCACACACCAGCAGGTTATC	

**Table 2:** LC–MS analysis of polyphenol extract of *Forsythia suspensa*.

Forecast Name	Formula	*m*/*z*	Retention Time	ppm	Sample 1	Sample 2	Sample 3
Homovanillic acid	C_9_H_10_O_4_	183.07	168.23	2.53	52700113.89	54429312.49	59345516
Hydroquinone	C_6_H_6_O_2_	110.02	764.41	0.05	1260209574	4236266335	5676053369
Isoproterenol	C_11_H_17_NO_3_	194.11	755.64	19.29	35845622.15	39613804.54	35094556.51
Norepinephrine	C_8_H_11_NO_3_	169.05	323.93	5.93	77926318	82202776.03	80226125.76
p-Synephrine	C_9_H_13_NO_2_	150.09	319.16	27.67	52490375.21	58003743.35	57080320.62
Sinapyl alcohol	C_11_H_14_O_4_	193.09	415.71	1.42	33910641	32486078.48	30376260.19
Tyrosol	C_8_H_10_O_2_	139.07	327.78	2.85	1272156773	1285278177	1147555904

## Data Availability

Data is contained within the article and supplementary materials.

## Supplementary Materials

The following supporting information can be downloaded at: https://www.mdpi.com/article/10.3390/antiox11030475/s1, Figure S1: Effect of Forsythia suspense extract on cell viability; Table S1: Differential metabolic.
