# Supplementary material for: Polyphenol Rich Forsythia suspensa Extract Alleviates DSS-Induced Ulcerative Colitis in Mice through the Nrf2-NLRP3 Pathway
Source: Antioxidants (Basel). 2022 Feb 28;11(3):475. doi: 10.3390/antiox11030475 (PMC8944444; doi:10.3390/antiox11030475)
Supplement: Supplementary file 1 [file antioxidants-11-00475-s001.zip › antioxidants-1593994-supplementary/Supplementary Materials/Figure S1.pdf]

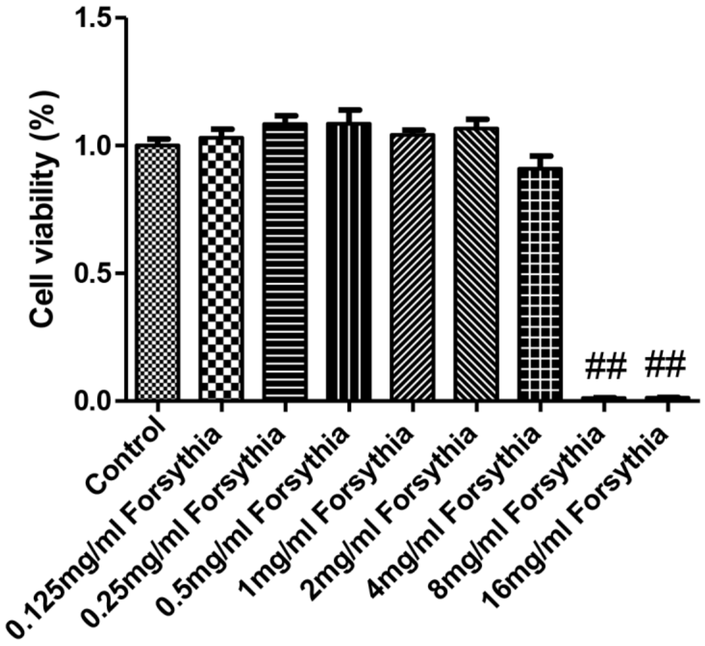

Figure S1 Effect of *Forsythia suspense* extract on cell viability. The results are shown as the mean ± SEM. ## P <0.01 compared with the control group
